# Brain Embolism Secondary to Cardiac Myxoma in Fifteen Chinese Patients

**DOI:** 10.1155/2014/718246

**Published:** 2014-03-09

**Authors:** Youming Long, Cong Gao

**Affiliations:** ^1^Key Laboratory of Neurogenetics and Channelopathies of Guangdong Province and The Ministry of Education of China, Institute of Neuroscience and the Second Affiliated Hospital of GuangZhou Medical University, 250 Changgang East Road, Guangzhou, Guangdong 510260, China; ^2^Department of Neurology, The Second Affiliated Hospital of GuangZhou Medical University, 250 Changgang East Road, Guangzhou, Guangdong 510260, China

## Abstract

*Background*. Heart myxoma-related embolisms commonly involve the central nervous system, but data are lacking in Chinese patients. *Methods*. 27 patients diagnosed with myxoma were reviewed retrospectively. *Results*. Among 27 patients, fourteen (51.9%) patients were women. Fifteen (55.6%) patients had brain embolisms. Rarely, patients were misdiagnosed with central nervous system vasculitis (*n* = 2), moyamoya disease (*n* = 1), and neuromyelitis optica (*n* = 1). We found positive associations between mRS (>3) and female gender (*r* = 0.873, *P* < 0.0001), infection (*r* = 0.722, *P* = 0.002), severe complications (*r* = 0.722, *P* = 0.002), systolic blood pressure (SBP) of <120 mmHg (*r* = 0.6, *P* = 0.018), WBC count of >10 × 10^9^/L (*r* = 0.722, *P* = 0.002), tumour size (*r* = 0.866, *P* < 0.0001), bilateral brain lesions (*r* = 0.60, *P* = 0.018), and total anterior circulation infarction (TACI) (*r* = 0.667, *P* = 0.007). The independent relationships among these factors and outcomes could not be confirmed (*P* > 0.05). *Conclusions*. Neurologic manifestations in Chinese patients with cardiac myxoma-related stroke were complicated and multifarious. Female gender, infection, other severe complications, low SBP, tumour size, bilateral brain lesions, TACI, and high WBC counts could be associated with a poor prognosis.

## 1. Introduction

Cardiac primary neoplasms are rare with an incidence of <0.2% at autopsy [1]. Among living patients who have undergone heart surgery, only 0.45% suffered from a heart tumour [2]. Myxomas, which are derived from multipotent mesenchymal cells of the endocardium [3], are the most common type of benign heart tumour, representing 50% to 83% of all primary cardiac tumours [2, 4, 5]. In recent years, increasingly more cases of cardiac myxoma [6] have been reported, including cases involving Chinese patients [5, 7].

The clinical manifestations of cardiac myxoma are generally nonspecific; a minority of patients are asymptomatic [6, 8]. The most common presentation is related to mitral valve obstruction and is characterised by palpitations, dizziness, dyspnoea, cough, heart failure, and syncope [6, 9]. The second most common presentation involves embolism formation, which may occur in any extremity but commonly involves the CNS [4, 6, 9, 10].

Many unique manifestations or therapies for CNS embolism secondary to cardiac myxoma have been reported. However, large case studies of patients with cardiac myxoma-related CNS embolism have not been published, although a few previous series have been reported [11–15]. Neurologic manifestations of atrial myxomas have been reported in 12% to 45% of affected patients [6, 8, 9, 11, 13, 14] and were quite various. The neurologic signs and symptoms are usually a result of emboli from a myxomatous or tumour-adherent thrombus. Cerebral embolic events often occur before the onset of constitutional or obstructive symptoms at a rate of up to 80% [12]. Other seldom seen neurological presentations occur in some patients, including intracerebral haemorrhage [14, 16], brain metastasis [17], and intracranial aneurysm [14].

Although atrial myxoma is associated with a high incidence of stroke, few patients die of cerebral embolism complications [17, 18]. No consistent, definitive prognosis has been established in these severely affected patients after stroke [19].

There are no published series that describe CNS manifestations and outcomes of cardiac primary tumours in Chinese patients with embolism. In the present study, we collected cases of Chinese patients affected by cardiac myxoma-related brain embolism and focused on the particular clinical characteristics of the embolisms.

## 2. Patients and Methods

27 patients with definite cardiac myxomas were evaluated by medical record reviews and interviews in the present study.

Data were retrospectively collected from patient records. At baseline, demographic data (age and sex) and history of conventional vascular risk factors were obtained. All patients with stroke underwent neurologic testing (brain CT or MRI, magnetic resonance angiography, or cerebral angiography) or neurologic consultation routinely within 24 to 48 hours after the attack. Laboratory investigations for vascular risk factors, duplex sonography of the carotid and vertebral arteries, and a thorough cardiac investigation were performed.

Cerebral infarctions were classified according to the OCSP systems [20]. The National Institute of Health stroke scale was used during the acute phase, and the modified Rankin Scale (mRS) was available and used in 15 patients with brain embolisms. The mRS score 3 months after onset was classified as independent (score of 0–2) or dependent/dead (score of 3–6).

Permission for the study was obtained from the Local Ethics Committees of the Second Affiliated Hospital of Guangzhou Medical University.

### 2.1. Statistical Analyses

Statistical analyses were performed with Fisher's exact test for binary and categorical data and the Mann-Whitney* U* test for continuous variables. Correlations between multiple variables and functional outcomes were analysed by Spearman's correlation test. Predictors of prognosis were analysed by binary logistic regression analysis, which allows for adjustment for confounding factors. Analyses were undertaken with the SPSS (version 11.1) software package.

## 3. Results

### 3.1. Demographic and Clinical Characteristics

Among the 27 patients with cardiac myxoma, the mean age was 57.3 ± 13.5 years (range, 34–85 years), and 14 (51.9%) patients were female. The left atrium (LA) was the most commonly observed location of cardiac myxomas (24 patients (88.9%)), followed by the right atrium (2 (7.4%)) and the LA and ventricles (1 (3.7%)).

The most common initial manifestation of myxoma in our series was brain embolism (15 patients (55.6%)), and the second was related to mitral valve obstruction: palpitations (7 patients (25.9%)) and Aase syndrome (1 patient (3.7%)). Three patients (11.1%) had no signs before surgery. Among the 25 patients with LA myxomas, 15 (60%) developed an embolism at the first attack.

### 3.2. Comparison of Patients with Cardiac Myxoma with and without Brain Embolism

The characteristics of these two groups of patients are shown in Table 1. No statistically significant differences in sex, age, and comorbidities were identified between the groups (*P* > 0.05). Patients with brain embolism were more likely to have infection on admission (*P* = 0.043). Table 1 also shows a statistically significantly higher white blood cell (WBC) count, complement 4 level, low-density lipoprotein level, and lactate dehydrogenase level in the patients with brain embolism on admission. Transthoracic echocardiography (TTE) results were available in all 27 patients. Fifteen (100%) patients with embolism had an irregular morphology compared with the patients without embolism (66.7%). However, other manifestations of TTE were not significantly different between the two groups. Logistic regression analysis showed that none of the factors showing significant differences (infection, complement 4 level, low-density lipoprotein level, creatine kinase MB level, lactate dehydrogenase level, WBC count, and irregular morphology) had predictive value for embolism formation (*P* > 0.05).

### 3.3. Clinical Features of Patients with Cardiac Myxoma with Brain Embolism

**Figure 1:**
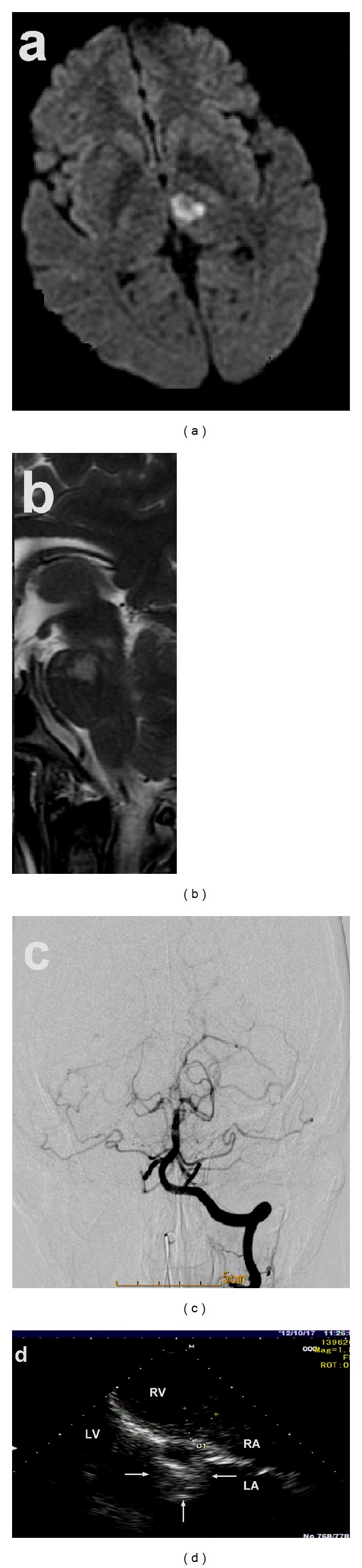
A patient presented with NMO-like attack. A 34-year-old woman with bilateral impaired vision, double vision, quadriplegia, dysuria, and internuclear ophthalmoplegia had normal brain CT results on 14 October 2012. Brain MRI showed multiple new lesions in brain. (a) MRI revealed abnormalities beside the third ventricle. (b) MRI revealed lesions in the pons. (c) DSA revealed occlusion of the top of the basilar artery. (d) Echocardiography showed a highly mobile mass attached to the fossa ovalis of the left atrium (arrow).

**Figure 2:**
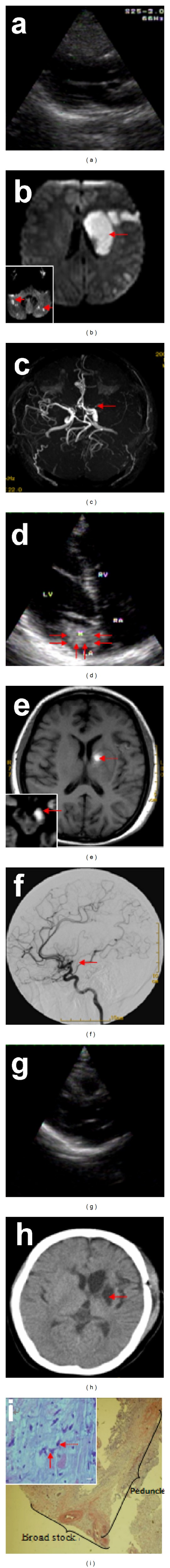
Patient presented with moyamoya disease. (a) Echocardiography with normal findings at the time of the first attack. (b) Brain MRI showed multiple new lesions in the left brain and bilateral cerebellum (arrow) at the first attack. (c) MRA revealed occlusion of the middle cerebral artery (arrow). (d) Echocardiography revealed a highly mobile mass attached to the fossa ovalis (arrow) at the second attack. (e) The second MRI showed a haematoma (dotted arrow) in the left basal ganglia and new infarction lesions in the left cerebral peduncle. (f) DSA showed occlusion of the middle cerebral artery (arrow) and secondary formation of an unusual vascular network (not shown). (g) After surgery, echocardiography showed that the mass in the left atrium had disappeared. (h) CT showed a stroke capsule in the left brain (arrow). (i) Histopathology showed spindle (dotted arrow) and stellate mesenchymal cells (arrow) that were embedded in the myxomatous stroma.

Ten (66.7%) patients had comorbidities (hypertension, hyperlipidemia, vascular malformation, hyperthyroidism, coronary heart disease, or atrial fibrillation). Motor deficits were seen in all patients with embolism at onset. Symptoms of conscious disturbance were the initial manifestation in 6 of 15 (40%) patients with embolism. Five of 15 (33.3%) patients had aphasia, and 3 (20%) had vertigo. Two (13.3%) patients had seizure attacks, and one had right vision loss because of central retinal artery occlusion. One patient had neuromyelitis optica-like manifestations at the onset of attack (Figure 1). One patient experienced two attacks and was diagnosed with CNS vasculitis and moyamoya disease (Figure 2). Embolism-related complications occurred in 10 of the 15 (66.7%) patients. Infection was the most common complication (8 patients (53.3%)). One patient died of sudden death. Two patients died of hernias after the embolism. Thus, the fatality rate was 20% (3 of 15) among patients with embolism. Only one patient had no symptoms 24 months after tumour resection.

**Figure 3:**
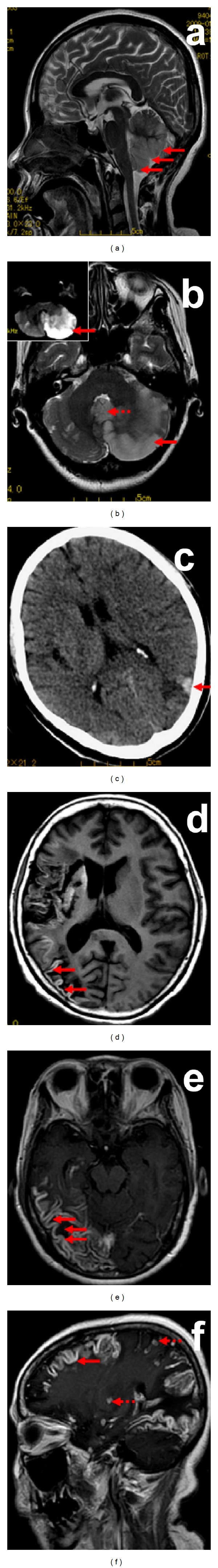
Unusual imaging findings in patients with myxoma with embolism. (a) A patient's death was due to the mass effect of a large infarction associated with compression. (b) Cross section of MRI revealed a new infarction of his left cerebellum (arrow). Part of the infarction compressed the fourth ventricle (dotted arrow). (c) CT showed a haematoma of the left cornu posterius ventriculi in the same patient (arrow). ((d)–(f)) MRI revealed a linear hyperintense band along the gyral margins (arrow) on T1-weighted imaging, which was defined as cortical laminar necrosis.

Neuroimaging was performed on all patients with embolism. Twelve of 15 (80%) patients had multiple cerebral infarcts; in 8 (53.3%), the infarctions involved the bilateral brain. Six (40%) patients had total anterior circulation infarction (TACI), and eight (53.3%) had partial anterior circulation infarction. Six (40%) patients had posterior circulation infarction, and five (33.3%) had anterior circulation infarction. The temporal lobe (*n* = 9), parietal lobe (*n* = 8), frontal lobe (*n* = 7), basal ganglia (*n* = 7), and cerebellum (*n* = 7) were the most commonly involved regions. No patients had spinal cord infarctions. Brain haemorrhage was seen in six (40%) patients. Not only did the location of the infarction differ but also its form differed among the 15 patients. Sporadic infarction occurred all in male patients, and multiple large lesions were found in most female patients (7 of 9 (77.8%)). Three (20%) patients had cortical laminar necrosis (CLN), an unusual MRI finding (Figure 3) [21].

### 3.4. Clinical Outcomes

At 3 months after onset, the mean mRS score of the patients with myxoma-related stroke was 3.34 ± 2.16 (range, 0–6). Six (40%) patients had a good outcome (mRS score of 0–2), and nine (60%) had a severe outcome (mRS score of 3–6). During the follow-up period (3–108 months), none of the 12 surviving patients experienced tumour recurrence or myxoma-related embolism.

Patients with severe outcomes (mRS score of 3–6) were almost all women (*P* = 0.001). They more often had infection (*P* = 0.011) and other severe complications (*P* = 0.011).

When Spearman correlation coefficients were calculated for prognosis and other variables, we found highly positive associations between high mRS scores (>3) and female gender (*r* = 0.873, *P* < 0.0001), infection (*r* = 0.722, *P* = 0.002), severe complications (*r* = 0.722, *P* = 0.002), systolic blood pressure (SBP) of <120 mmHg (*r* = 0.6, *P* = 0.018), WBC count of >10 × 10^9^/L (*r* = 0.722, *P* = 0.002), tumour length + width of >5 cm (*r* = 0.866, *P* < 0.0001), bilateral brain lesions (*r* = 0.60, *P* = 0.018), and TACI (*r* = 0.667, *P* = 0.007). However, on stepwise multiple logistic regression analysis, the independent relationships among these factors (infection, severe complications, WBC count, TACI, and admission SBP) and severe outcomes could not be confirmed (Table 2).

## 4. Discussion

Our female/male ratio (14/13) in cardio myxomas differs from that of most other studies, in which this ratio varies from 1.7 to 4.4 : 1 [6, 22], including a large sample from China [7]. Only one Chinese study showed results consistent with ours; in that study, 39 of 75 patients with cardiac myxomas were women [5]. Whether this female predominance is correlated with the pathogenesis of cardiac myxoma is unclear. One previous report indicated that gender is associated with neurologic or embolic symptoms in men and systemic symptoms in women [6]. In our series, we did not find that female gender was associated with embolism when comparing the embolic and nonembolic groups, which is similar to the results of a series in Korea [13]. In terms of gender, the present results could be of major interest for patients with brain embolism. Indeed, there was an obvious female predominance in worse outcomes (mRS score of >3). Although it cannot be considered an independent predictor of poor outcome in this small sample, it had a high correlation with poor prognosis (*r* = 0.873; *P* < 0.0001). In our opinion, female patients with myxomas should be offered close monitoring and early surgery because of the possibility of a worse outcome, including death.

The data in our study could be of major interest in terms of disease manifestation in the 15 patients with embolism. First, most myxoma-related emboli occluded large arteries or multiple vascular territories; eight (53.3%) patients had multiple infarctions in the bilateral brain, and six (40%) had total anterior circulation infarction. Our occurrence rate of multiple infarctions in the bilateral brain was higher than that in previous studies (3 of 11 in Korea [13] and 0 of 9 in the United States [14]). This indicates that our patients with cardiac myxoma with embolism had more severe injuries. Second, patients had special manifestations that had not been reported in previous studies. Neuromyelitis optica (NMO) may sometimes appear similar to top-of-the-basilar syndrome [23]. But in the present study, a patient, who had pathologically confirmed myxoma-related embolism, presented with NMO-like manifestation (Figure 1). And there were other two patients who had a prior presumptive diagnosis of CNS vasculitis before a definitive diagnosis was reached, especially a woman also with moyamoya disease, which has never been reported (Figure 2). Third, unusual MRI findings in patients with myxoma-related embolism are another interesting factor [14]. In our study, 20% of patients had CLN. CLN represents neuronal ischemia accompanied by gliosis and layered deposition of fat-laden macrophages [24].

Complications after ischemic stroke are common, and they may adversely impact the clinical outcome. Although comprising a small sample, our patients had a high complication rate of 73.3% (11 of 15 patients). Herniation (*n* = 2), haemorrhagic transformation (*n* = 6), and epilepsy (*n* = 2) were the cerebral complications found in seven patients, six (85.7%) of whom had a poor outcome. In a previous study, poor outcomes and death were more frequent in patients with cerebral embolism of cardiac origin [25, 26], similar to our present results. In the present study, two patients died of transtentorial herniation and cerebellar tonsillar herniation, respectively. Herniation and death seldom occurred in patients with myxoma in several previous series [11–14], but death was reported in a single case report [27]. The most severe cardiac complication of myxomas is sudden death. Sudden death secondary to primary cardiac tumours is very infrequent and limited to single case reports in the literature. A review indicated that there were 120 cases (approximately 0.0025%) of sudden death attributed to primary cardiac tumours from 1982 to 1991 [28]. In the present study, one was a case of sudden death, the rarest complication of heart tumour. She was suddenly unresponsive, and her presenting rhythm was asystole. To our knowledge, this is the first report of a patient with sudden death secondary to LA tumour after brain embolism. This case indicates the importance of early surgery to decrease such unusual death.

Infection was the most common complication among our patients with cardiac myxoma with brain embolism (9 of 15 patients (75%)). Previous studies on acute stroke have demonstrated that pneumonia and urinary tract infection are independently associated with poor outcomes or increase the risk of death [29, 30]. Although stepwise multiple logistic regression analysis did not show infection as an independent predictor of poor outcomes in our small sample, it was highly related to poor prognosis (*r* = 0.722; *P* = 0.002). Therefore, given the higher infection and mortality rates in our patients compared with other causes of stroke, prophylactic antibiotic treatment is necessary in patients with severe embolism secondary to heart myxoma.

There are certain limitations to our study. Because of the retrospective nature of the study, uncontrolled or unknown factors that could affect the outcome might have confounded our results. It is also possible that retrospective identification of patients might have caused selection bias. The sample of 15 patients from a single centre in our hospital was still small. However, to the best of our knowledge, the present study represents the largest clinical series of brain embolism related to cardiac myxoma, and it is helpful for the clinician.

In summary, most patients with myxoma-related embolism had complications and poor outcomes. Female gender, infection, complications, low SBP, tumour size, bilateral brain lesions, TACI, and WBC counts could be associated with the outcome. We hope that a large-sample, multicentre, prospective evaluation will be performed in our Chinese patients with myxoma-related embolism.

## Figures and Tables

**Table 1:** Clinical features of patients with cardiac myxoma with brain embolism.

Characteristic	Total	Embolism	No embolism	*P*
	(*n* = 27)	(*n* = 15)	(*n* = 12)	
Female/male	12/15	9/6	6/6	0.863
Age, y (mean ± SD)	57.3 ± 13.5	56.4 ± 13.3	58.4 ± 14.1	0.708
Hypertension, *n* (%)	9 (33.3%)	6 (40%)	3 (25%%)	0.683
Hyperlipidemia, *n* (%)	5 (18.5%)	4 (26.5%)	1 (8.3%)	0.342
Vascular malformation, *n* (%)	2 (7.4%)	2 (13.3%)	0	0.487
Diabetes at baseline, *n* (%)	2 (7.4%)	0	2 (16.7%)	0.188
Coronary artery disease, *n* (%)	3 (11.1%)	2 (13.3%)	1 (8.3%)	1
Atrial fibrillation, *n* (%)	5 (18.5%)	3 (20%)	2 (16.7%)	1
Infection, *n* (%)	8 (29.6%)	7 (46.7%)	1 (8.3%)	0.043
Death, *n* (%)	4 (14.8%)	3 (20%)	1* (8.3%)	0.605
Complement 4, g/L (mean ± SD)	0.32 ± 0.08	0.35 ± 0.09	0.27 ± 0.06	0.036
Low-density lipoprotein, mmol/L (mean ± SD)	2.72 ± 0.84	3.08 ± 0.71	2.23 ± 0.78	0.017
Creatinine kinase-MB, U/L (mean ± SD)	12.75 ± 9.96	16.64 ± 11.66	8.0 ± 4.39	0.05
Lactate dehydrogenase, U/L (mean ± SD)	247.48 ± 178.38	316.42 ± 208.51	155.56 ± 55.94	0.037
Hydroxybutyrate dehydrogenase, U/L (mean ± SD)	188.85 ± 162.94	246.45 ± 202.77	118.44 ± 39.08	0.078
Platelet count, ×10^9^/L (mean ± SD)	270.2 ± 100.9	286.2 ± 119.6	249.9 ± 70.9	0.383
Red blood cell count, ×10^9^/L (mean ± SD)	4.35 ± 0.61	4.55 ± 0.51	4.08 ± 0.15	0.054
White blood cell count, ×10^9^/L (mean ± SD)	10.3 ± 3.9	11.6 ± 4.4	8.6 ± 2.3	0.049
Echocardiography Features				
Myxomas length cm (mean ± SD)	4.31 ± 1.68	4.04 ± 1.21	4.65 ± 2.14	0.233
Attached to atrial septum, *n* (%)	24 (88.9%)	14 (93.3%)	10 (83.3%)	0.569
High echo, *n* (%)	22 (81.5%)	12 (80%)	10 (83.3%)	1
Irregular shape, *n* (%)	23 (85.2%)	15 (100%)	8 (66.7%)	0.028
Peduncle, *n* (%)	21 (77.8%)	11 (73.3%)	10 (83.3%)	0.662
Prolapse into valve, *n* (%)	22 (81.5%)	14 (93.3%)	8 (66.7%)	0.139
Aortosclerosis, *n* (%)	17 (63.0%)	10 (66.7%)	7 (58.3%)	0.371
Aortic valve regurgitation, *n* (%)	10 (37.0%)	5 (33.3%)	5 (41.7%)	0.706
Mitral valve regurgitation, *n* (%)	12 (44.4%)	7 (46.7%)	5 (41.7%)	1
Tricuspid valve regurgitation, *n* (%)	15 (55.6%)	7 (46.7%)	8 (66.7%)	0.441
Lower left ventricular compliance, *n* (%)	10 (37.0%)	7 (46.7%)	3 (25%)	0.424
Heart enlargement, *n* (%)	13 (48.1%)	6 (40%)	7 (58.3%)	0.449

*One patient with right atrium myxoma and patent foramen ovale died of the complication of surgery.

**Table 2:** Features of different outcomes of patients with myxoma with brain embolism.

Characteristic	MRS 0–2 (*n* = 6)	MRS 3–6 (*n* = 9)	*P*
Female/male	0/6	8/1	0.001
Age, y (mean ± SD)	55.44 ± 14.20	57.83 ± 10.54	0.748
Infection, *n* (%)	1 (16.7%)	8 (88.9%)	0.011
Other severe complications*, *n* (%)	1 (16.7%)	8 (88.9%)	0.011
Hemorrhagic transformation, *n* (%)	1 (16.7%)	5 (55.6%)	0.287
Accompanying disease, *n* (%)	3 (50%)	7 (77.8%)	0.329
Systolic blood pressure, mm Hg (mean ± SD)	135 ± 9.61	116.89 ± 13.71	0.032
Diastolic blood pressure, mm Hg (mean ± SD)	86.2 ± 11.07	71.89 ± 12.53	0.058
Conscious disturbance, *n* (%)	0	6 (66.7%)	0.028
MRI			
Bilateral lesions, *n* (%)	1 (16.7%)	7 (77.8%)	0.041
Lesions count (mean ± SD)	2.17 ± 1.21	4 ± 1.33	0.026
Total anterior circulation, *n* (%)	0	6 (66.7%)	0.028
Posterior circulation, *n* (%)	1 (16.7%)	5 (55.6%)	0.287
WBC, ×10^9^/L (mean ± SD)	8.21 ± 3.18	13.52 ± 3.52	0.029
Hemoglobin, g/L	139.4 ± 20.27	117.4 ± 14.89	0.053
MCH pq (mean ± SD)	29.96 ± 2.31	26.29 ± 3.21	0.059
MCHC, g/L (mean ± SD)	339.2 ± 6.62	320.3 ± 13.35	0.022
IgM (mean ± SD)	0.61 ± 0.18	1.22 ± 0.38	0.023
Echocardiography			
ROVT, cm (mean ± SD)	2.97 ± 0.23	2.60 ± 0.25	0.010
AO, cm (mean ± SD)	3.24 ± 0.03	2.78 ± 0.22	0.026
Myxomas length cm (mean ± SD)	2.89 ± 1.39	4.6 ± 0.87	0.009
Myxomas (length + width) cm (mean ± SD)	4.93 ± 2.27	8.0 ± 2.18	0.017
